# A new role for human dyskerin in vesicular trafficking

**DOI:** 10.1002/2211-5463.12307

**Published:** 2017-09-12

**Authors:** Nunzia Di Maio, Rosario Vicidomini, Alberto Angrisani, Valentina Belli, Maria Furia, Mimmo Turano

**Affiliations:** ^1^ Department of Biology University of Naples ‘Federico II’ Napoli Italy; ^2^ Present address: NICHD (National Institute of Child Health and Human Development)‐ Section on Metabolic Regulation ‐NIH‐ 35 Convent DR Bethesda MD USA; ^3^ Present address: Medical Oncology Department of Clinical and Experimental Medicine “F. Magrassi” Universitá degli Studi della Campania “Luigi Vanvitelli” Naples Italy

**Keywords:** cytoskeletal remodeling, *DKC1*, Rab5/Rab11 endosomes

## Abstract

Dyskerin is an essential, conserved, multifunctional protein found in the nucleolus, whose loss of function causes the rare genetic diseases X‐linked dyskeratosis congenita and Hoyeraal‐Hreidarsson syndrome. To further investigate the wide range of dyskerin's biological roles, we set up stable cell lines able to trigger inducible protein knockdown and allow a detailed analysis of the cascade of events occurring within a short time frame. We report that dyskerin depletion quickly induces cytoskeleton remodeling and significant alterations in endocytic Ras‐related protein Rab‐5A/Rab11 trafficking. These effects arise in different cell lines well before the onset of telomere shortening, which is widely considered the main cause of dyskerin‐related diseases. Given that vesicular trafficking affects many homeostatic and differentiative processes, these findings add novel insights into the molecular mechanisms underlining the pleiotropic manifestation of the dyskerin loss‐of‐function phenotype.

**Abbreviations**
ALT: alternative lengthening of telomeres
DAPI: 4′‐6‐diamidino‐2‐phenylindole
Dox: doxycycline
Dxr: doxorubicin
ERC: endosomal recycling compartment
FACS: fluorescence‐activated cell sorting
GAPDH: glyceraldehyde 3‐phosphate dehydrogenase
GSS: glutathione synthetase
GTPase: guanosine triphosphate hydrolase
HH: Hoyeraal‐Hreidarsson
IRESs: internal ribosome entry sites
miRNAs: microRNAs
MTOC: microtubule‐organizing center
MTT: 3‐(4,5‐dimethylthiazol‐2‐yl)‐2,5‐diphenyltetrazolium bromide
MVBs: multivesicular bodies
PARP: poly (ADP‐ribose) polymerase
qRT: quantitative real‐time reverse transcription
Rab11A: Ras‐related protein Rab‐11A
Rab5: Ras‐related protein Rab‐5A
shRNA: short hairpin RNA
snoRNAs: small nucleolar RNAs
snoRNPs: small nucleolar RNA ribonucleoproteins
Tet: tetracycline
WGA: wheat germ agglutinin
X‐DC: X‐linked dyskeratosis congenita

Vesicular transport is a fundamental way of communication between the cell and its microenvironment and regulates many vital cellular processes, including internalization of several types of molecules, nutrients uptake, membrane protein turnover, cell adhesion or migration properties, and receptor signaling 1. A variety of molecules shuttle in and out of cells via the endocytic/exocytic pathways. Along the endocytic pathway, the cargo is internalized by either clathrin‐dependent or clathrin‐independent pathways and then routed to sorting endosomes, to be subsequently addressed to lysosomes for degradation, or returned back to membrane via either the fast or the late‐recycling routes. The Ras‐related in brain (Rab) small G proteins are master regulators of vesicular trafficking 2, 3. In particular, Ras‐related protein Rab‐5A (Rab5)‐Rab4 sorting endosomes have a key role in mediating the fast recycling to membrane, while Rab11 endosomes mediate the slow recycling, moving from the endocytic recycling compartment (ERC), localized near the centrosomal microtubule‐organizing center (MTOC), to the cell surface 4, 5. Dysregulation of vesicle trafficking can underlie diverse aspects of cancer cell biology, including loss of cell polarity, transformation, invasion, and metastasis, so that aberrant expression/regulation of Rab guanosine triphosphate hydrolase (GTPases), including Rab5 6 and Rab11 7, has been associated with tumorigenesis 8.

Here, we report that the ubiquitous nucleolar protein dyskerin, a component of the small nucleolar ribonucleoprotein complexes (snoRNPs), plays an unexpected role in the regulation of vesicle trafficking. Dyskerin, encoded by the human *DKC1* gene, is a multifunctional highly conserved protein that participates in diverse nuclear ribonucleoprotein complexes, such as those of active telomerase, type H/ACA snoRNPs, and small Cajal ribonucleoproteins 9. All these complexes are involved in a variety of crucial biological functions that include safeguarding telomere integrity, ribosome biogenesis, and pseudouridylation of cellular RNAs 10, 11, 12. In addition, dyskerin has been shown to act as a cotranscriptional factor of key pluripotency‐related genes in mammalian embryonic stem cells 13. Considering the variety of these biological functions, it is not surprising that *DKC1* hypomorphic mutations cause hereditary disorders, respectively, known as X‐linked dyskeratosis congenita (X‐DC) and Hoyeraal‐Hreidarsson (HH) syndrome 14. Main manifestation of these diseases is a triad of mucocutaneous features accompanied by chronic bone marrow failure, telomere instability, premature aging, and increased susceptibility to various types of cancers 15, 16. Although many authors consider X‐DC and HH mainly as telomeropathies, a wide bulk of data support the alternative view that the primary cause of these diseases can be associated with telomerase‐independent roles of dyskerin. For example, *DKC1* ^*m*^ mice show symptoms of the disease before a telomere shortening is detectable 15. Similarly, in an X‐DC zebrafish model, changes in telomerase activity were undetectable at early stages, supporting the view that telomerase deficiency is not responsible for the onset of X‐DC pathogenesis 17. In addition, although Drosophila lacks a canonical telomerase, Drosophila dyskerin is essential for fly viability and its depletion causes a large variety of developmental defects 18, 19, 20, 21. Finally, snoRNPs have recently gained an important role in several pathologies, including cancer 22, 23.

To investigate in more detail the primary effects triggered by dyskerin loss‐of‐function phenotype, we generated colon carcinoma (RKO) and osteosarcoma (U2OS) stable cell lines expressing a short hairpin RNA (shRNA) able to trigger inducible silencing of the *DKC1* gene. These cellular systems enabled us to analyze in detail the cascade of events occurring within a short time frame (1–3 cell doublings) immediately following dyskerin knockdown, and thus largely preceding the time needed for telomere shortening. With this approach, we found that dyskerin downregulation quickly causes cytoskeletal remodeling and dysregulation of Rab5‐Rab11 vesicular trafficking, thus revealing additional and unexpected mechanisms by which this protein can affect cell homeostasis.

## Materials and methods

### Construction of the inducible pLKO‐Tet‐On‐shDKC1 silencing vector

To insert sequences encoding *DKC1* shRNA, the pLKO‐Tet‐On plasmid (Novagen, Cambridge, MA, USA) was digested with *Age*I and *Eco*RI enzymes (Promega, Madison, WI, USA) and then annealed and ligated with two partially overlapping oligonucleotides targeting the junction of *DKC1* 12–13 exons. Oligonucleotide sequences carrying *Age*I and *Eco*RI sites at their ends were as follows: forward, 5′ CCGGTATGTTGACTACAGTGAGTCTCTCGAGAGACTCACTGTAGTCAACATATTTTT 3′; reverse, 5′AATTAAAAATATGTTGACTACAGTGAGTCTCTCGAGAGACTCACTGTAGTCAACATA 3′ (Sigma‐Aldrich, St. Louis, MO, USA). Competent Stbl4 *Escherichia* cells (Invitrogen, Carlsbad, CA, USA) were transformed with ligation products, and plasmid DNA from positive clones were sequenced to confirm the identity of the sh*DKC1* silencing construct, hereafter named pLKO‐Tet‐On‐sh*DKC1*.

### Cell culture and generation of shRNA‐expressing stable cell lines

RKO and U2OS human cell lines were obtained from ATCC (Manassas, VA, USA) and cultured as previously described 24. To generate stable cell lines, cells were transfected with 3 μg pLKO‐Tet‐On‐sh*DKC1* and 12 μL of Metafectene Pro (Biontex Laboratories GmbH, München, Germany), following the manufacturer's instructions. After 20 days of puromycin selection (750 ng·mL^−1^; Sigma‐Aldrich), independent clones were collected and maintained in media supplemented with Tet‐free FBS (Clontech Laboratories Inc, Mountain View, CA, USA) and puromycin. In the absence of tetracycline (Tet), or its synthetic derivative doxycycline (Dox), shRNA*DKC1* expression is repressed by the binding of the constitutively expressed TetR protein to the Tet‐responsive element; Tet/Dox addition (400 ng·mL^−1^) to the medium triggers shRNA expression, resulting in targeted *DKC1* silencing 25.

### Cell proliferation assays and cytoskeletal analyses

To measure cell vitality and proliferation, an equal number of RKO Dox‐treated and untreated cells (3 × 10^5^/dish) were seeded in triplicate in 100‐mm plates, harvested every 24 h up to 4 days, stained with 0.5% trypan blue (Euroclone spa, Milan, Italy), and counted by a Burker chamber. For 3‐(4,5‐dimethylthiazol‐2‐yl)‐2,5‐diphenyltetrazolium bromide (MTT) assay, Dox‐treated and untreated cells were seeded in triplicate at the density of 5 × 10^3^ cells per well in 96‐well plates. The day after, culture medium was aspirated and, after a washing in PBS, replaced with 100 μL of 0.5 mg·mL^−1^ MTT solution per well. After 4‐h incubation at 37 °C in 5% CO_2_ incubator, the medium was removed and the precipitated formazan was dissolved in 100 μL of acidic isopropanol. The absorbance was quantified by spectrophotometry at 570 nm using the microplate reader Victor3 Multilabel Counter (Perkin Elmer, Waltham, MA, USA).

To trigger cytoskeletal disruption, cells were incubated in medium containing 3 μm methyl [5‐(2‐thienylcarbonyl)‐1H‐benzimidazol‐2‐yl] (nocodazole; Sigma‐Aldrich) or 3 μm latrunculin A (Sigma‐Aldrich) for 2 h at 37 °C and subsequently analyzed by confocal immunofluorescence microscopy (see below).

### FACS analysis

Control and Dox‐treated cells were trypsinized at the indicated time, counted, washed three times in PBS, and fixed in ice‐cold methanol at −20 °C overnight. Cells were then washed twice with cold PBS, counted, and rehydrated at the density of 10^6^ cells·mL^−1^ by incubation in PBS for 30 min on ice. Subsequently, cells were suspended in hypotonic solution 0.1% Na‐citrate, 50 μg·mL^−1^ RNase, 50 μg·mL^−1^ propidium iodide and incubated for 30 min in the dark at room temperature. The DNA content was measured using a fluorescence‐activated cell sorting (FACS) Calibur flow cytometer (Becton Dickinson, Franklin Lakes, NJ, USA), and data were analyzed using the cell quest pro and modfit3.0 software packages (Becton Dickinson).

### RNA and protein analysis

Total RNA extraction, preparation of first‐strand cDNA, and quantitative real‐time reverse transcription (qRT)‐PCR were carried out as previously described 26 Oligonucleotide sequences were as follows: *DKC1*‐F 5′‐GGCGGATGCGGAAGTAAT‐3′, *DKC1*‐R 5′‐CCACTGAGACGTGTCCAACTT‐3′, glutathione synthetase‐F (*GSS*‐F) 5′‐GGACTGGCCCTGGGAATT‐3′, *GSS‐*R 5′‐CCTTCTCTTGAGCAATCAGTAGCA‐3′. Western blot analysis was conducted according to 27. Used antibodies are reported in Table S1.

### WGA and transferrin uptake assays, immunofluorescence analysis

For wheat germ agglutinin (WGA) live staining, cells were incubated at room temperature for 10 min with WGA/Texas red conjugate (W21405; Thermo Fisher Scientific, Waltham, MA, USA) and fixed with 3.7% paraformaldehyde for 10 min. For confocal immunofluorescence analysis, RKO‐ and U2OS‐transfected cells were preliminarily seeded on glass coverslips in six‐well plates and treated for 72 h with Dox. Dox‐treated or untreated cells were then fixed with 3.7% paraformaldehyde for 10 min, permeabilized in 0.3% Triton X‐100 for 15 min, and blocked in PBS supplemented with 3% BSA for 30 min. After each step, the cells were rinsed in PBS, incubated for 1 h at room temperature with primary antibodies and then for 30 min at room temperature with secondary antibodies (listed in Table S1). Uptake assay of Alexa Fluor 488‐conjugated transferrin (T13342; Thermo Fisher Scientific) was conducted according to 28. Upon indicated treatments, coverslips were mounted on glass slides with Hoechst solutions and examined under the fluorescence confocal microscope Zeiss LSM 700 (Zeiss, Oberkochen, Germany).

## Results

### Generation and validation of stable cellular systems for *DKC1*‐inducible knockdown

**Figure 1:**
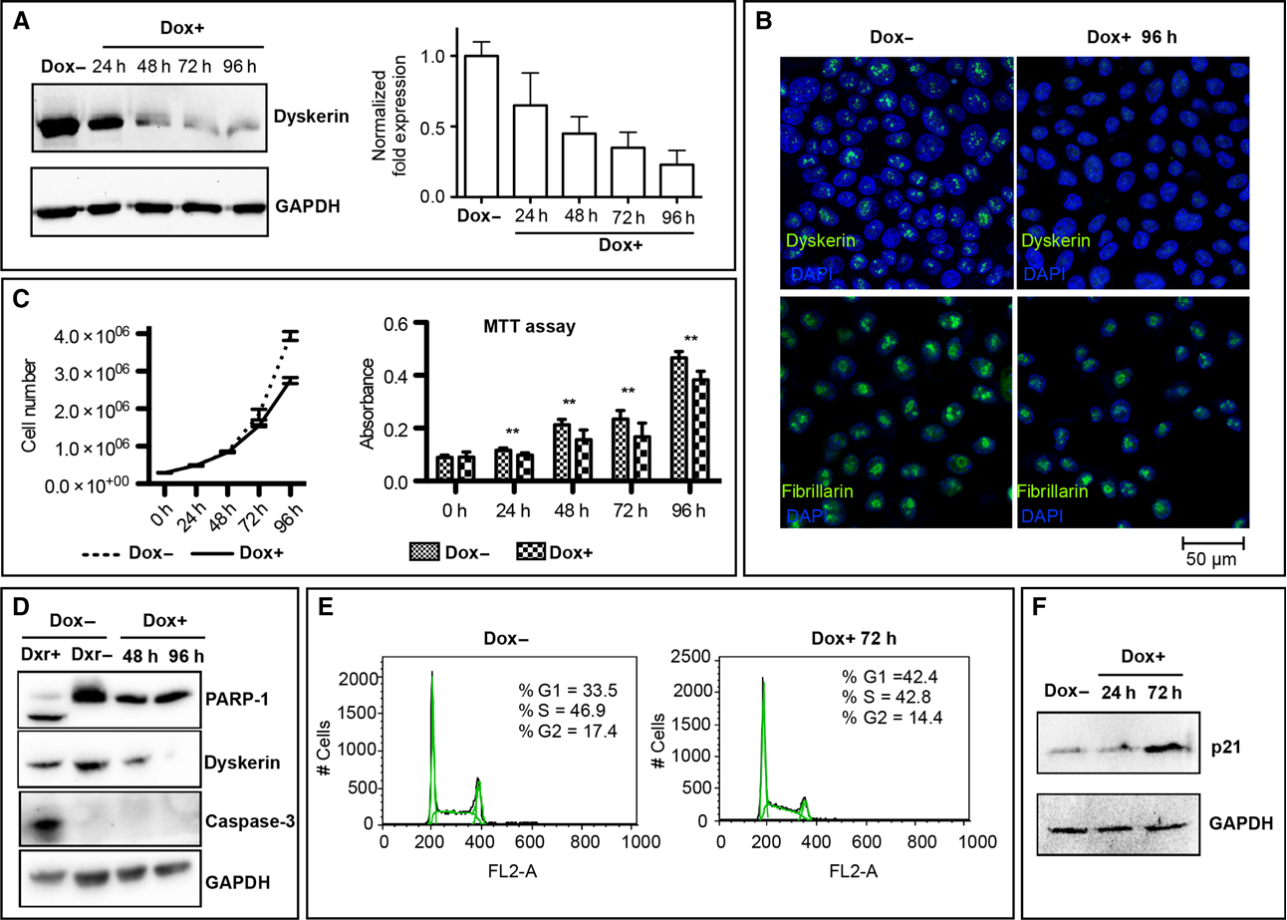
Validation of Dox‐inducible *DKC1* silencing and its effects on proliferation of RKO cells. (A) Time‐course analysis of *DKC1* expression upon Dox‐induced silencing: on the left, immunoblotting analysis of dyskerin levels; on the right, qRT‐PCR analysis of dyskerin mRNA accumulation. (B) Confocal microscopic images of control (Dox−) and silenced (Dox+) RKO cells confirming the efficacy of dyskerin depletion and the appropriate nucleolar localization of fibrillarin. The sum of total z‐stack focal planes is shown. (C) On the left, growth curves of control (Dox−) and silenced cells (Dox+); on the right, MTT assay: a two‐way ANOVA test was performed, with **P < 0.005. For both experiments, each point represents the mean ± SEM of three different experiments. (D) No activation of the PARP1 and caspase‐3 apoptotic markers was observed in dyskerin‐depleted (Dox+) cells, while both markers were induced upon Dxr treatment (Dxr+), used as positive control (first lane). These results indicate that the reduction in cell number was not due to apoptosis. (E) FACS analysis indicates that dyskerin‐depleted cells (Dox+) are delayed at G1/S progression compared to their control (Dox−). (F) Western blot analysis displayed an increase in p21 expression in the silenced cells, confirming that G1/S progression is halted (in agreement with FACS results).

To perform a careful analysis of the early events triggered by dyskerin depletion, we generated an inducible *DKC1* silencing lentiviral vector that contained all the necessary components to express *DKC1* shRNA upon Tet/Dox addition (see Materials and methods). The silencing efficiency of the vector, named pLKO‐Tet‐On‐sh*DKC1*, was first tested in the RKO human colon cancer cells that are diploid, poorly differentiated, and express wild‐type p53, APC, and β‐catenin 29. Independent stable clones were isolated and *DKC1* knockdown evaluated at both mRNA and protein levels. As shown in Fig. 1A, the system was quickly responsive, as dyskerin amount was significantly reduced after only 24 h from Dox induction and further decreased subsequently. In strict agreement, reverse qPCR experiments showed a parallel downregulation of dyskerin mRNA levels. Confocal microscopic analysis confirmed a strong reduction in dyskerin accumulation in the nucleoli of the silenced cells (Fig. 1B), further validating the silencing efficiency. Moreover, the silencing conditions did not perturb the nucleolar accumulation of fibrillarin, a highly conserved nucleolar protein associated with box C/D small nucleolar RNAs (snoRNAs) 30, leading to rule out the occurrence of obvious nucleolar alterations (Fig. 1B). Altogether, these observations showed that this inducible system was able to generate stable cellular clones useful to define the early consequences of dyskerin depletion. A set of different approaches was then performed and followed on independent clones. First, we checked the effects of *DKC1* gene silencing on cell proliferation. Dyskerin depletion has in fact generally been reported to perturb this parameter 31, 32, although the specific phase of the cell cycle at which cells accumulate diverged among different cell types. Consistent with previous findings, both a direct count of viable cells and MTT measurements indicated that cell proliferation progressively decreased upon Dox‐induced *DKC1* silencing (Fig. 1C). We confirmed that the decrease in cell number was not due to apoptosis, as poly (ADP‐ribose) polymerase‐1 (PARP‐1) and caspase 3, two known apoptotic markers, were not activated upon gene silencing. Both markers were instead efficiently cleaved upon treatment with the proapoptotic topoisomerase II inhibitor doxorubicin (Dxr), thereby providing a positive control (Fig. 1D). FACS analysis revealed an increase in the G1 percentage of the silenced cells (42.4% compared to 33, 5% of control cells; Fig. 1E), accompanied by a slight reduction in the S‐phase (42.8% compared to 46.9% of the control cells) and G2‐phase percentages (14.4% compared to 17.4% of control cells). Dyskerin depletion was reported to induce a block at G1 also in yeast 33 and, more recently, in neuroblastoma cells 34; in this latter case, the proliferative arrest was observed to be unrelated to human telomerase RNA levels or to telomerase activity 34, 35. To further investigate the effect of dyskerin depletion on cell cycle, we checked the expression of p21, a protein that plays a key role in G1/S cell cycle arrest 36. In keeping with FACS results, p21 accumulation raised significantly upon dyskerin depletion (Fig. 1F), confirming that G1/S progression was halted. Noticeably, this effect occurred very early upon dyskerin knockdown and thus had no correlation with telomere instability.

### Dyskerin depletion affects Rab5‐ and Rab11‐mediated recycling

**Figure 2:**
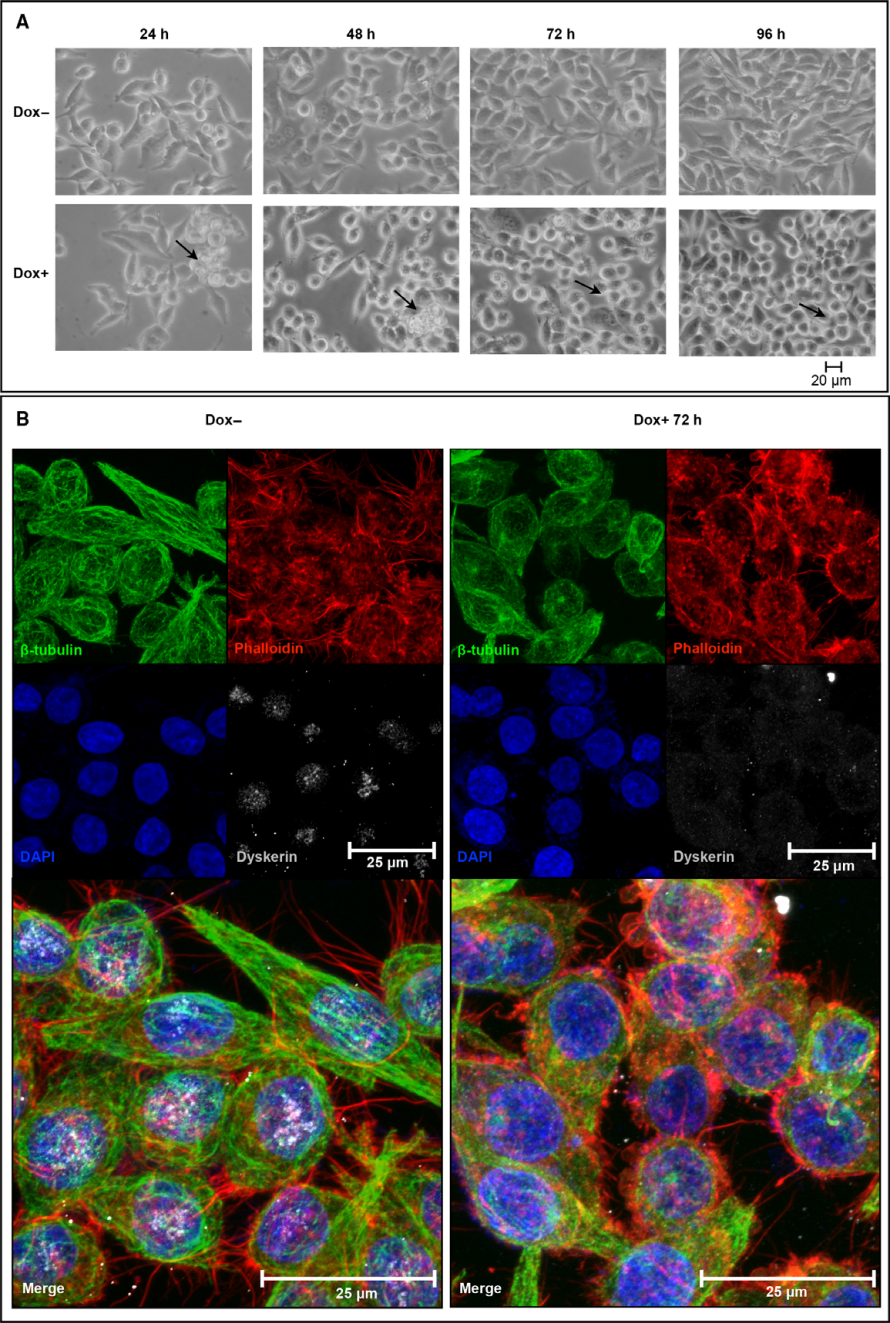
Dyskerin‐depleted RKO cells exhibit morphological changes and cytoskeletal rearrangement. (A) Phase‐contrast imaging of control (Dox−) and silenced cells, the latter treated with Dox for 72 h (Dox+). Note that, upon Dox induction, the cells tend to assume a round‐shaped morphology (see black arrows). (B) Confocal analysis of control (Dox−) and silenced cells (Dox+) upon costaining with β‐tubulin (green) and dyskerin (gray) antibodies and phalloidin (red). Nuclei were counterstained with 4′‐6‐diamidino‐2‐phenylindole (DAPI; blue). The sum of five z‐stack central focal planes is shown.

**Figure 3:**
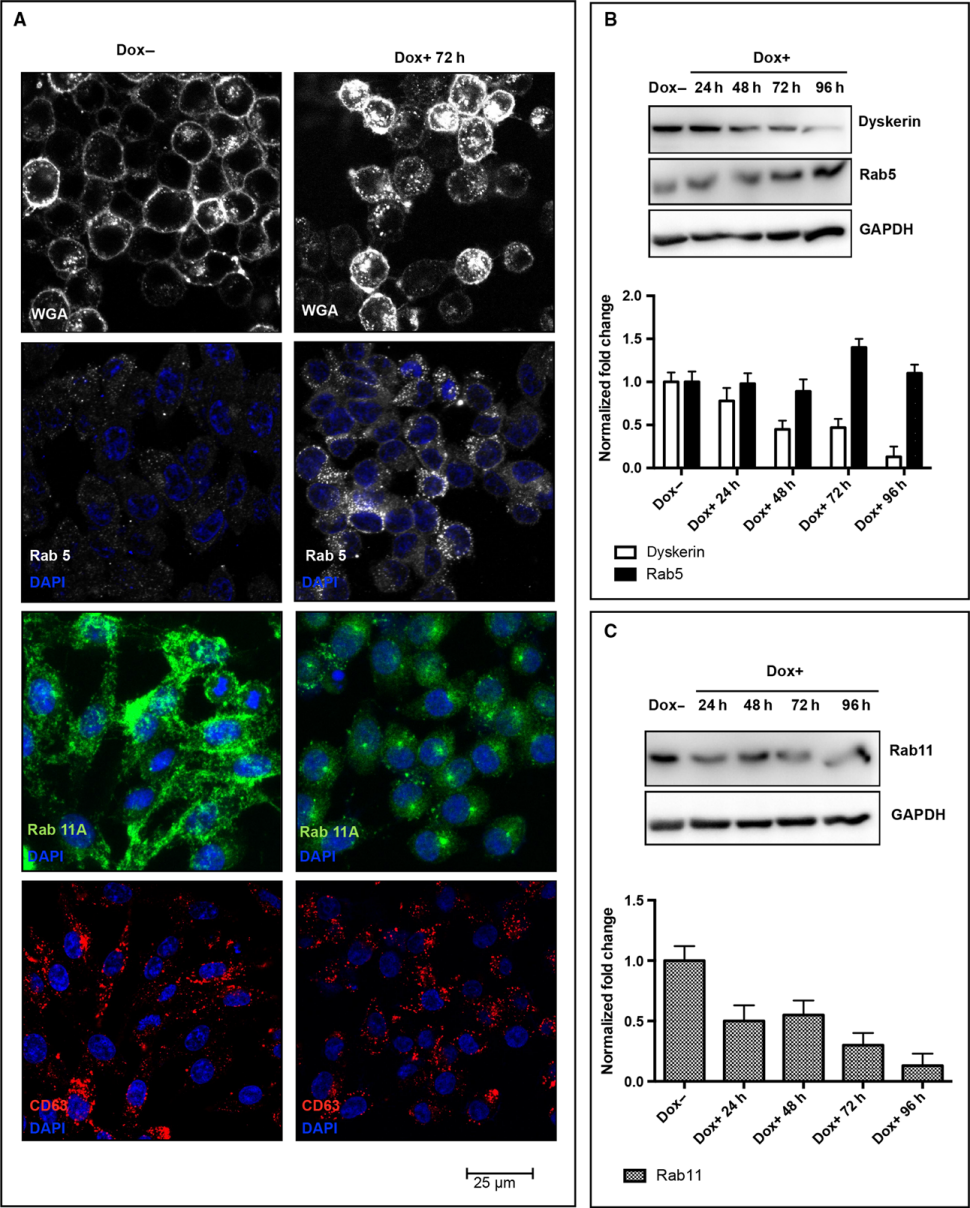
Dyskerin‐depleted RKO cells show enhanced endocytosis. (A) Confocal analysis of control (Dox−) and silenced cells (Dox+) upon WGA uptake *in vivo* (top), or upon staining with Rab5, Rab11A or CD63 (bottom) antibodies. Images of control and silenced cells were acquired at the same conditions. For WGA and Rab11A analysis, the image shows the sum of z‐stack total focal planes; for Rab5 and CD63 analysis, images show the sum of five z‐stack central focal planes; nuclei were counterstained with 4′‐6‐diamidino‐2‐phenylindole (DAPI). Note that an increase in WGA active uptake and Rab5‐mediated vesiculation characterize dyskerin‐depleted cells, while no significant alteration in the distribution of CD63 vesicles is observed. (B) Immunoblotting time‐course analysis of Rab5 expression upon Dox‐induced dyskerin silencing and relative densitometric quantitative analysis. (C) Immunoblotting time‐course analysis of Rab11A expression upon Dox‐induced dyskerin silencing and relative densitometric quantitative analysis.

Morphological analysis of RKO‐silenced cells showed that only 24 h from silencing induction they lost the typical epithelia‐like structure and tended to assume a round‐shaped morphology and appeared highly refractile when observed by phase‐contrast microscopy (Fig. 2A). In addition, about 10% of these cells detached from the plate. However, when recruited from the medium, they remained unstained by the trypan blue dye and, after transferring in a Dox‐free medium, recovered their substratum‐adhesive property (data not shown). These observations suggested that this feature was reversible and dependent on *DKC1* gene activity, and highlighted that dyskerin depletion can promote a cytoskeletal remodeling resulting in a quick transition from an adherent state to a suspended one. Thus, we first analyzed the microtubule and actin networks by immunofluorescence staining of β‐tubulin and F‐actin. Indeed, analysis of confocal images indicated that, upon dyskerin depletion, RKO cells are characterized by reduced anti‐β‐tubulin immunoreactivity and presence of less stretched and oriented microtubule filaments. In addition, phalloidin staining revealed that the actin meshwork appeared thinner and displayed a reduced number of filopodia in the silenced cells (Fig. 2B). These observations fully support the view that cytoskeletal scaffolding undergone a prompt rearrangement. As microtubules and actin filaments, together with motor proteins, support vesicles movements 1, we wondered whether cytoskeletal remodeling had any impact on vesicular trafficking. To gain information on this latter aspect, we firstly followed the *in vivo* internalization of Texas red‐conjugated WGA, a carbohydrate‐binding lectin that recognizes sialic acid and *N*‐acetylglucosaminyl sugar residues on the plasma membrane. WGA is widely used to follow receptor‐mediated membrane transport and vesicular trafficking; its uptake is an active, energy‐dependent process mediated by both actin filaments and microtubules 37. As shown in Fig. 3A, WGA staining marked more heavily both the peripheral membrane and the internal vesicles of dyskerin‐depleted cells, suggesting an increase in the receptor‐mediated endocytic process. To further explore this feature, we analyzed by confocal microscopy the distribution of vesicles positive for Rab5, a small GTPase that marks the early Rab4‐Rab5 endosomes and mediates the fast recycling to membrane 2. Indeed, Rab5 staining incremented significantly in the silenced cells (Fig. 3A), supporting the conclusion that dyskerin depletion can enhance endocytosis and the fast recycling route. To check the slow recycling of endocytosed proteins, by which the cargo traverses the endosomal recycling compartment (ERC) and is addressed back to cell periphery, we looked at the Rab11 late‐recycling endosomes 5. Confocal microscopic analysis of distribution of Rab11 vesicles showed that in the control cells they mainly concentrated at ERC and at cortical periphery, where they are particularly enriched at cell–cell contacts (Fig. 3A). In contrast, in dyskerin‐depleted cells, the density of Rab11 endosomes appeared drastically reduced, with these vesicles essentially clustered at pericentrosomal ERC, poorly diffused throughout the cytoplasm, and nearly absent at cell periphery (Fig. 3A). The loss of Rab11 cortical localization indicated that the slow recycling to membrane was drastically hampered in the silenced cells, implying a possible dysregulation of this important process 38. Worth noting, western blot analyses confirmed that Rab5 and Ras‐related protein Rab‐11A (Rab11A) expression levels are differentially influenced by dyskerin depletion (Fig. 3B,C). As Rab11 vesicles play a regulatory role also in the exocytic process, modulating the transport of secretory vesicles to the plasma membrane 39, we labeled cells with CD63, a marker of both late endosomes lysosomes and exocytic multivesicular bodies (MVBs) 40. However, no significant alteration in the density/distribution of CD63 vesicles, or in their mobilization toward cell periphery, was observed (Fig. 3A), suggesting that dyskerin depletion specifically affected the dynamics of Rab11‐mediated transport from ERC to the membrane. To ensure that Dox treatment could not elicit, by itself, any of the above‐described effects, we treated RKO‐untransfected cells with 400 ng·mL^−1^ Dox for 72 h. As shown in Fig. S1, Dox treatment *per se* did not induce changes in cell shape, cytoskeletal remodeling, or alteration in density/localization of Rab5/Rab11 endosomes, confirming the specificity of the observed phenotypes.

### Rab11 mislocalization in dyskerin‐depleted cells is not due to the disruption of cytoskeletal scaffolding

**Figure 4:**
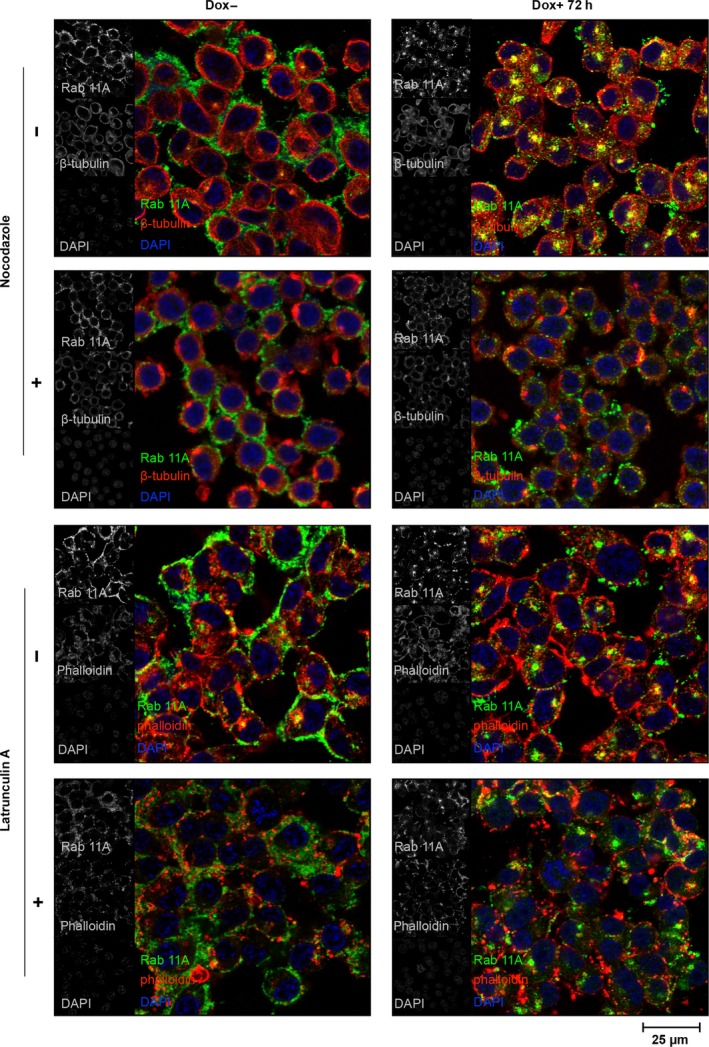
Dyskerin‐depleted RKO cells are characterized by reduced density and mislocalization of Rab11 vesicles. Confocal images of control (Dox−) and silenced cells (Dox+), untreated (−) or treated (+) with nocodazole (top) or latrunculin A (bottom). Cells were costained with Rab11A (green) and β‐tubulin (red) antibodies or with Rab11A antibody (green) and phalloidin (red). Nuclei were counterstained with 4′‐6‐diamidino‐2‐phenylindole (DAPI; blue). All signals are shown in gray in the left insets. Images of control and silenced cells were acquired at the same conditions; the sum of five z‐stack central focal planes is shown. Note that silenced cells are characterized by a dramatic reduction in Rab11A labeling at cell periphery, accompanied by a pericentrosomal accumulation at the ERC; this phenotype is not mimicked by cytoskeletal disruptions induced by either nocodazole or latrunculin A treatment, as both of them promote diffusion of Rab11 vesicles.

To rule out the possibility that Rab11 mislocalization could be due to cytoskeletal damages eventually caused by dyskerin depletion, we pretreated cells with nocodazole or latrunculin A, two cytoskeletal disruptive drugs, and then looked at the intracellular distribution of Rab11 endosomes. Treatment either with nocodazole, which rapidly depolymerizes microtubules and MTOC 41, or with latrunculin A, which depolymerizes F‐actin 42, 43, completely dispersed Rab11 vesicles from the pericentrosomal region, promoting their cytosolic diffusion in both control and silenced cells (Fig. 4). Cytosolic dispersion appeared more pronounced upon latrunculin treatment, suggesting that in RKO cells the Rab11 vesicles might travel preferentially along actin network. Anyhow, none of these treatments mimicked the Rab11 mislocalization observed upon dyskerin depletion. On the contrary, both drugs counteracted the accumulation of Rab11 vesicles at ERC, ruling out the possibility that this specific phenotype could be attributed to disruption of the cytoskeletal scaffolding. Collectively, the above results lead to conclude that dyskerin depletion can induce alterations in cell shape, cytoskeletal scaffolding, and vesicular trafficking within only 72 h from silencing induction. As none of these effects occurred upon Dox exposure of untransfected RKO control cells (Fig. S1), it is reasonable to conclude that they are specifically triggered by dyskerin depletion.

### Cytoskeletal remodeling and alteration of vesicular trafficking are telomerase‐independent effects of dyskerin depletion

**Figure 5:**
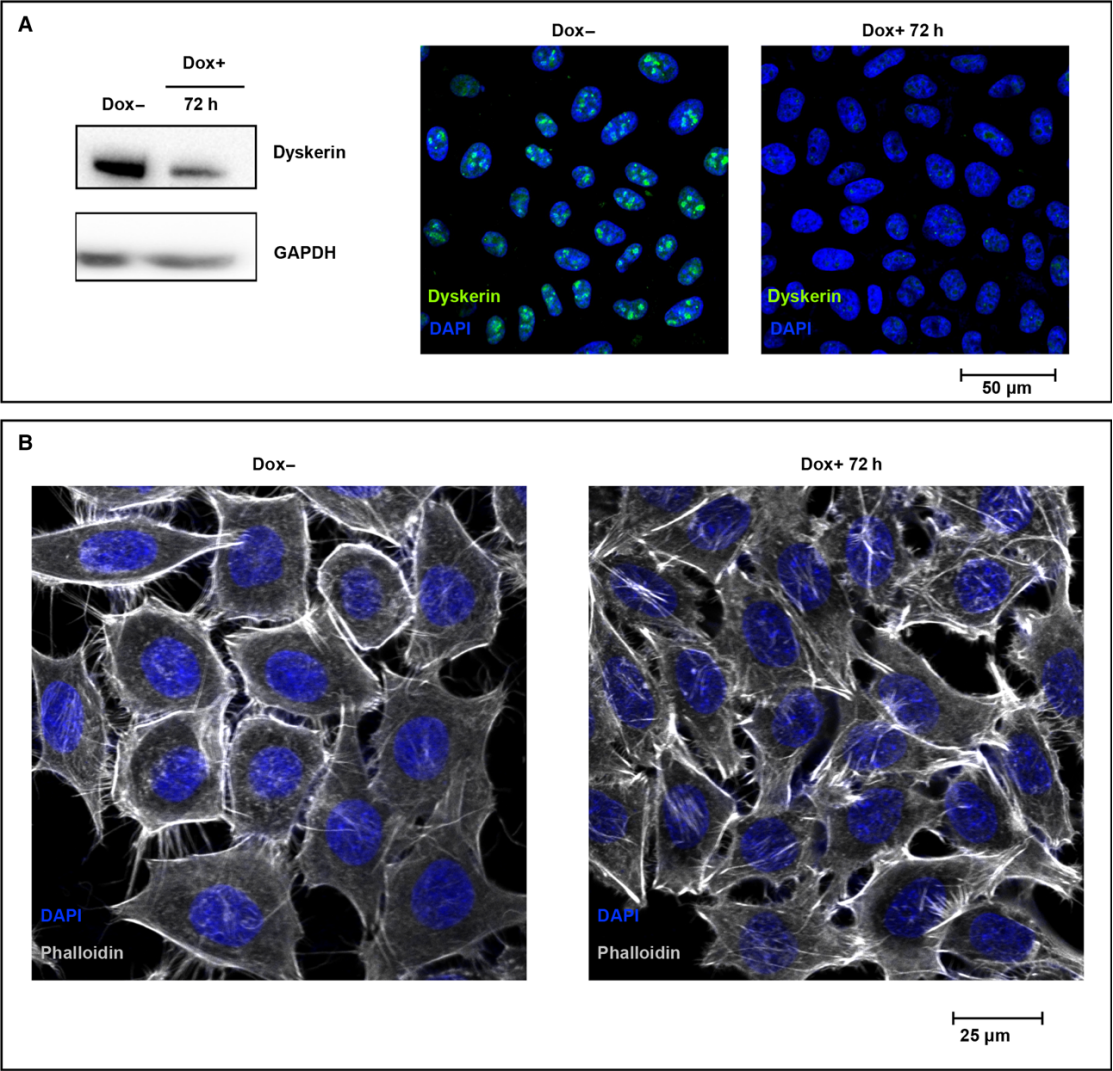
Morphological changes and cytoskeletal rearrangements upon *DKC1* silencing in the telomerase‐negative U2OS cells. (A) Western blot (left) and confocal immunofluorescence analysis (right) confirmed the efficient dyskerin downregulation upon 72 h of Dox induction. (B) Confocal analysis of U2OS control (Dox−) and silenced cells (Dox+) stained with DyLight 550 phalloidin; nuclei were counterstained by 4′‐6‐diamidino‐2‐phenylindole (DAPI; blue). Images of control and silenced cells were captured under the same conditions; a z‐stack central focal plane is shown. As observed in the RKO line, upon dyskerin depletion U2OS cells exhibit morphological changes, assuming a more extended shape.

**Figure 6:**
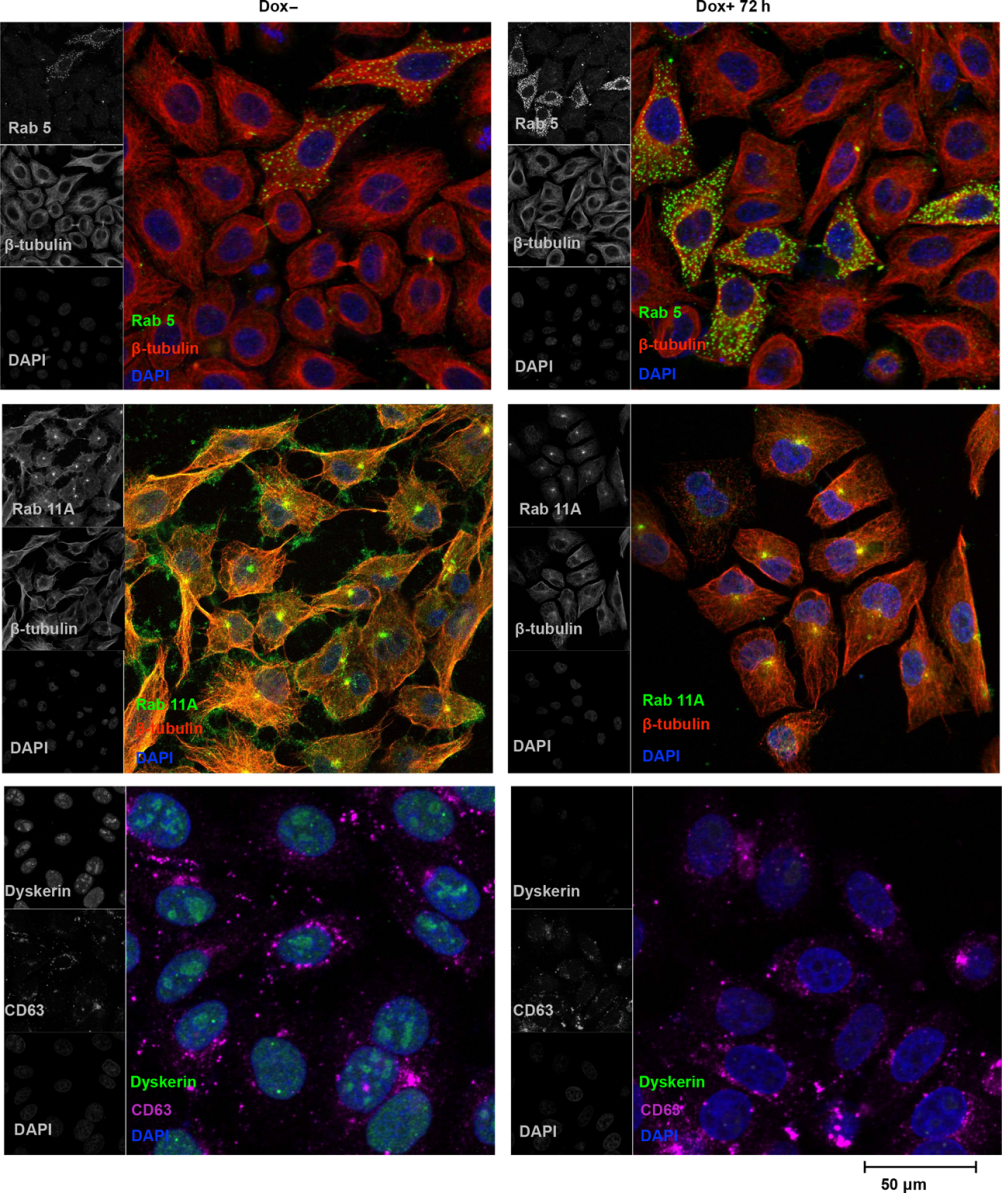
Dynamics of endosomal trafficking in U2OS dyskerin‐depleted cells. Confocal images of control (Dox−) and silenced cells (Dox+) costained with Rab5 (green) and β‐tubulin (red) antibodies (top); Rab11A (green) and β‐tubulin (red) antibodies (middle); dyskerin (green) and CD63 (magenta) antibodies (bottom). All signals are in gray in the left insets; nuclei were counterstained with 4′‐6‐diamidino‐2‐phenylindole (DAPI; blue). Images of control and silenced cells were captured under the same conditions; the sum of z‐stack five central focal planes is shown. As in RKO cells, dyskerin depletion enhances Rab5 trafficking while it hampers Rab11 recycling to membrane; no significant alteration in the distribution of CD63 vesicles is instead noticed.

**Figure 7:**
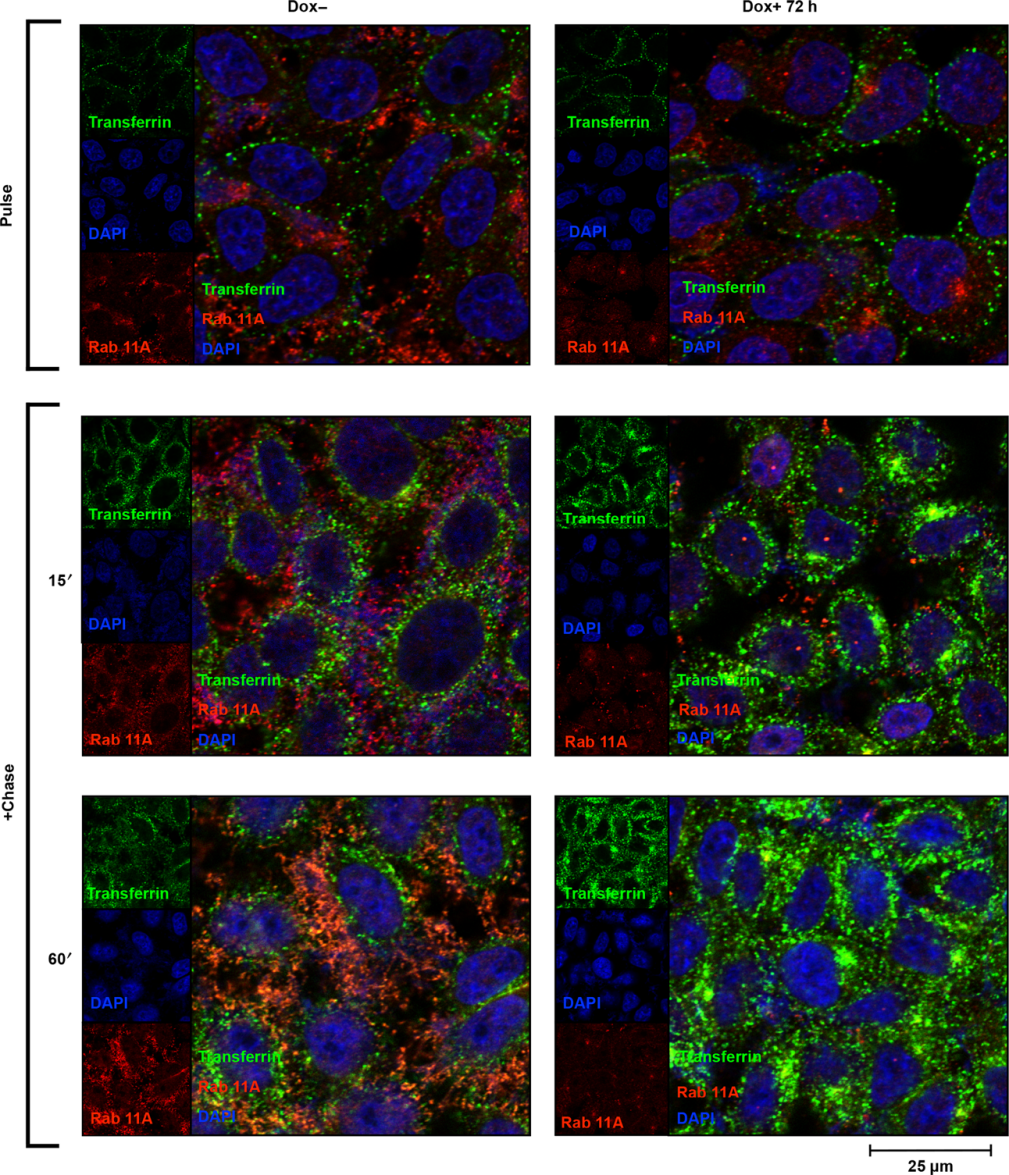
Transferrin uptake and its distribution in dyskerin‐depleted U2OS cells. Control (Dox−) and silenced (Dox+) U2OS cells were pulsed with Alexa Fluor‐488‐conjugated transferrin at 4 °C for 15 min (top), then chased at 37 °C for 15 or 60 min and stained with Rab11A antibody (red). Nuclei were counterstained with 4′‐6‐diamidino‐2‐phenylindole (DAPI; blue). A z‐stack central focal plane is shown. In both control and silenced cells, transferrin is efficiently internalized, as evident upon 15‐min chase (middle). However, upon 60‐min chase, transferrin colocalizes diffusely with the Rab11 vesicles, amply dispersed in the cytoplasm only in the control cells. Transferrin–Rab11A colocalization is scarce and restricted to some ERC regions in the dyskerin‐depleted cells (bottom).

To exclude cell line‐specific effects and to firmly assess that the above‐described effects were independent from telomere instability, we extended our analyses to the human osteosarcoma epithelial (U2OS) cell line. U2OS cells were selected because, although telomerase‐negative 32, they elongate telomeres efficiently by the alternative lengthening of telomeres (ALT) pathway 44. As shown in Fig. 5, the pLKO‐Tet‐On‐sh*DKC1* vector caused a very efficient dyskerin downregulation also in U2OS cells, confirming its general applicability. Remarkably, dyskerin depletion was again accompanied by an abrupt change in cell morphology, with U2OS‐silenced cells quickly assuming a more stretched shape (Fig. 5). Next, we checked the distribution of Rab5 and Rab11 endosomes. As shown in Fig. 6, Rab5 trafficking significantly increased upon dyskerin depletion, confirming the tendency to a more active endocytosis and to a fast recycling. The intracellular distribution of Rab11 slow‐recycling endosomes was also significantly altered, being even more evident in these cells because of their large size. In fact, in the control cells, the Rab11 vesicles heavily marked both ERC and cell periphery and were amply dispersed throughout the cytosol, where they closely matched the microtubule network (Fig. 6). In contrast, upon dyskerin depletion, the density of these late endosomes was strongly reduced and they appear predominantly clustered at ERC, poorly dispersed in the cytosol, and dramatically reduced at the cortical region (Fig. 6). To functionally analyze the endocytic and recycling processes, we next followed the internalization of transferrin. This protein is internalized by a receptor‐mediated process 45 and is recycled through the ERC by Rab11 vesicles 46. In these experiments, we incubated control and silenced cells with a 15‐min pulse of Alexa Fluor 488‐conjugated transferrin at 4 °C, followed by a chase of 15 and 60 min at 37 °C. As shown in Fig. 7, transferrin was very efficiently internalized also in dyskerin‐depleted cells where, immediately following its uptake (15‐min chase), assumed an intracellular distribution very similar to that observed in the control cells. In fact, in both silenced and control cells, transferrin did not colocalize with Rab11 at these early times. However, upon 60 min of chase, in the control cells a large amount of transferrin colocalized with Rab11 vesicles (Pearson's coefficient 0.8), which appeared amply dispersed throughout the cytoplasm. Conversely, in the silenced cells, transferrin and Rab11 signals colocalized essentially only at the ERC (Fig. 7), confirming the occurrence of a defective late recycling. Hence, the results of this functional assay further supported the conclusion that alteration of Rab11 receptor‐mediated recycling occurs in diverse cell lines as a precocious effect of dyskerin depletion.

## Discussion

To deeply investigate the range of dyskerin biological roles, we set up cellular systems able to trigger inducible *DKC1* gene silencing and allow a detailed, short‐term analysis of the events immediately following protein knockdown. Here, we report that dyskerin suppression shortly induces a cytoskeletal remodeling which triggers changes in cell shape well before the eventual occurrence of telomere erosion. Specifically, the cytoskeletal rearrangements induced by dyskerin depletion weakened cell adhesion to substrate in both RKO and U2OS cells, although this effect was less intense in the U2OS line, whose cells have thicker stress fibers and more robust focal adhesion complexes 47. Noticeably, dyskerin downregulation was found to induce loss of cell–substratum adhesion also in prostate carcinoma 31 and in neuroblastoma cells 32, although this aspect has remained so far poorly investigated. Indeed, it is reasonable to suppose that anchorage weakening can be a general feature that contributes to growth and proliferative impairment that characterizes various cell types upon dyskerin depletion. The reshuffling of the cytoskeleton is linked to many dynamic cellular processes, such as cell division, motility, endocytosis, and vesicular trafficking. Here, we focused our analysis on the intracellular transport, mainly with respect to endocytic and recycling processes. We found that the fast endocytic recycling was incremented in the silenced cells, possibly accelerating the return of specific cargos to membrane. At the same time, a dramatic reduction in Rab11 late‐recycling endosomes was observed, with these vesicles essentially clustered at the pericentrosomal ERC and nearly absent at cell cortex. Traveling of Rab11 vesicles from ERC to cortex requires interaction with a number of effectors, including the Rab11 family‐interacting proteins (FIPs), which mediate association with microtubule‐ or actin‐based molecular motors and enable both movement and correct intracellular positioning of these vesicles 4. The most obvious explanation for Rab11 accumulation at the ERC may thus rely on a transport defect. However, this phenotype has similarly been observed upon depletion of the Rab11 GAP Evi5 48, suggesting that also deregulated Rab11 activation can favor trapping of these vesicles at the ERC, strongly reducing Rab11‐dependent, slow recycling. This finding further highlights the complexity of dyskerin cellular functions, although the specific mechanisms by which this protein can influence cytoskeletal and vesicular dynamics remain elusive. Indeed, several telomerase‐independent roles of dyskerin may be involved. Dyskerin, by associating with other core proteins and H/ACA snoRNAs, can in fact participate in diverse ribonucleoprotein complexes involved in rRNA processing and site‐specific pseudouridylation of rRNA, snRNAs, as well as of mRNAs 49. In turn, reduction in rRNA pseudouridylation affects ribosome translation fidelity 50 and internal ribosome entry site‐dependent translation efficiency 51. Thus, it is conceivable that these functions could contribute to cytoskeletal rearrangement and alteration of vesicular trafficking. For example, dyskerin depletion may deregulate the expression of mRNAs involved in these processes, or affect these cellular dynamics in association with specific H/ACA snoRNAs. Intriguingly, this hypothesis is favored by recent studies that established an emerging role of snoRNAs in specific metabolic functions. A striking example is that of U17, a dyskerin binding snoRNA involved in intracellular cholesterol trafficking 52. Worth noting, proper sorting and trafficking within endosomal vesicles is necessary to maintain cellular homeostasis and to perform both ubiquitous and cell type‐specific functions. In fact, perturbation of this traffic widely affects cargo destination and membrane properties, this way potentially altering cell–cell and cell–extracellular matrix interactive communication and related differentiative events. Consistent with these premises, dyskerin depletion may cause both cell‐autonomous and nonautonomous effects, as observed for developmental defects and alterations of long‐range signaling occurring in Drosophila upon *in vivo* silencing of the *DKC1* orthologue 19, 20, 21. Finally, the endocytic pathway intersects other intracellular transport routes, such as the secretory pathway and the retrograde transport of selected cargo from ERC to the trans‐Golgi network (TGN). While the relationship with the TGN pathway remains to be investigated, the unaltered intracellular distribution of CD63 exocytic vesicles suggests that their specific trafficking is not affected. Collectively, our results indicate that *DKC1* silencing can perturb several aspects of cell homeostasis independently of telomere instability. The observation that dyskerin can orchestrate vesicular traffic not only adds further information on the multiple biological roles played by this protein but gives novel insights into the comprehension of molecular mechanisms underlying the congenital diseases triggered by dyskerin depletion.

## Author contributions

MT planned and, with NDM, participated in most experiments. VB took care of cell culture and western blot analyses. AA carried out MTT, FACS, and qRT‐PCR analyses. RV participated in confocal analyses and provided useful suggestions and critical advice. MF planned and coordinated the study, analyzed data, and wrote the manuscript with advice from MT.

## Supporting information


**Fig. S1**. Dox treatment *per se* did not elicit morphological changes or alteration in Rab5/Rab11 trafficking in RKO and U2OS wt cells.

**Table S1**. List of antibodies used in immunofluorescence (IF) and/or western blotting (WB) analyses.
