# Supplementary figures and images for: A new role for human dyskerin in vesicular trafficking
Source: FEBS Open Bio. 2017 Sep 12;7(10):1453–68. doi: 10.1002/2211-5463.12307 (PMC5623704; doi:10.1002/2211-5463.12307)

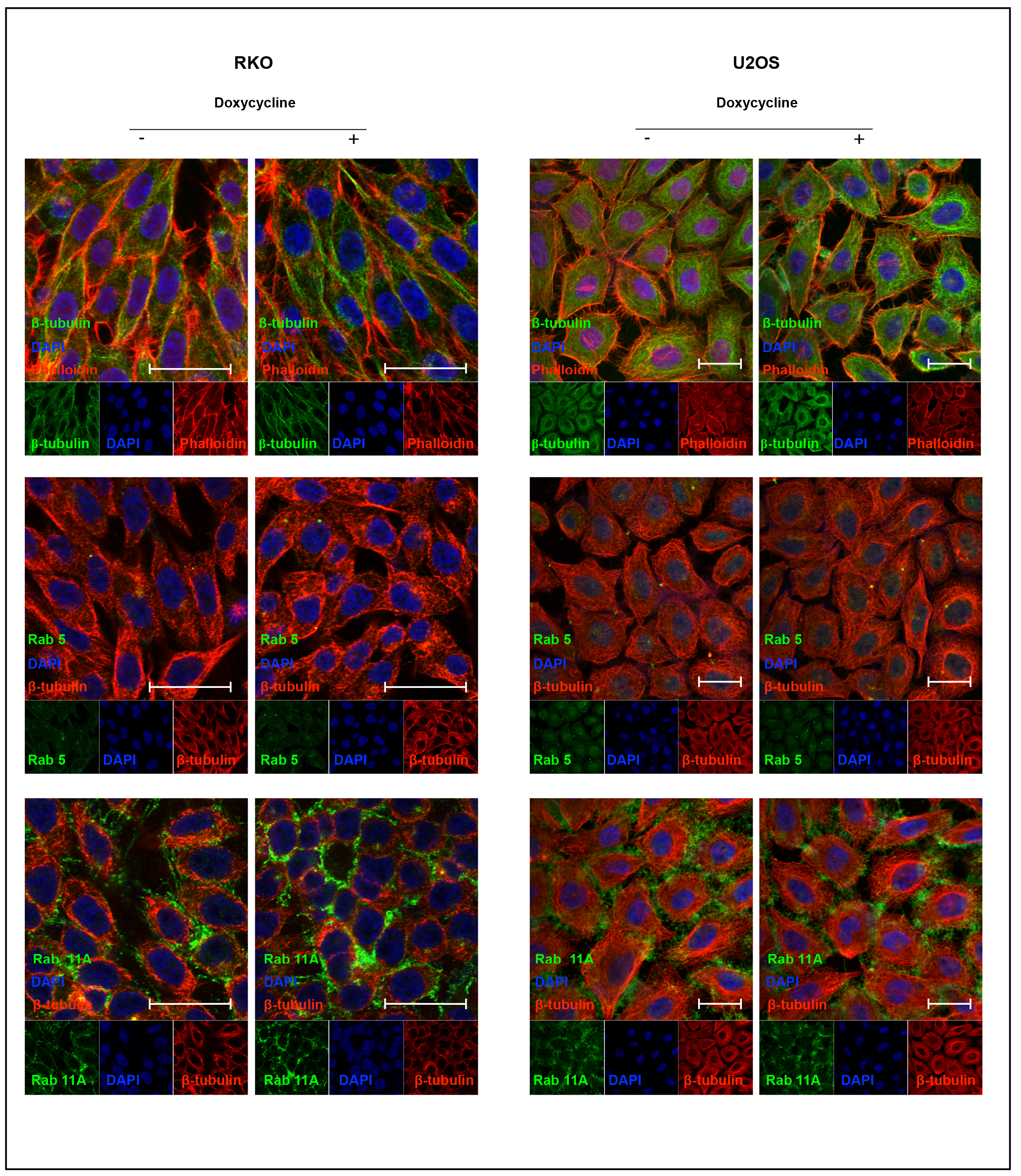

Supplement: Supplementary file 1 — Fig. S1. Dox treatment per se did not elicit morphological changes or alteration in Rab5/Rab11 trafficking in RKO and U2OS wt cells. [file FEB4-7-1453-s001.tif]
