# Supplementary material for: A new role for human dyskerin in vesicular trafficking
Source: FEBS Open Bio. 2017 Sep 12;7(10):1453–68. doi: 10.1002/2211-5463.12307 (PMC5623704; doi:10.1002/2211-5463.12307)
Supplement: Supplementary file 2 — Table S1. List of antibodies used in immunofluorescence (IF) and/or western blotting (WB) analyses. [file FEB4-7-1453-s002.pdf]

ANTIBODY	MANUFACTURER	CATALOGUE NUMBER	APPLICATION
**anti-dyskerin**	Santa Cruz Biotechnology, Dallas, TX, USA	sc-48794	WB/IF
**anti-PARP 1**	Santa Cruz Biotechnology, Dallas, TX, USA	sc-7150	WB
**anti-GAPDH**	Origene, Rockville, MD, USA	TA308884	WB
**anti-p21**	Origene, Rockville, MD, USA	TA307018	WB
**anti-caspase 3**	Millipore, Billerica, MA, USA	04-439	WB
**anti-Rab 11 A**	Abcam, Cambridge, UK	ab65200	WB/IF
**anti-Rab 5**	Santa Cruz Biotechnology, Dallas, TX, USA	ab18211	WB/IF
**anti-CD63**	Abcam, Cambridge, UK	ab8219	IF
**anti-β-tubulin**	Hybridoma bank, Iowa City, USA	E7-s	IF
**Cy3-conjugated anti-goat IGG**	Bethyl, Montgomery, USA	A50-201C3	IF
**FITC-conjugated anti-rabbit IGG**	Bethyl, Montgomery, USA	A120-100F	IF
**anti-rabbit**	Bethyl, Montgomery, USA	A120-100P	WB
**DyLight 550 Phalloidin**	Thermo Fisher Scientific, Waltham, MA, USA	21835	IF
